# Pharmacological Management of Transthyretin Amyloid Cardiomyopathy: Where We Are and Where We Are Going

**DOI:** 10.3390/jcm14103481

**Published:** 2025-05-16

**Authors:** Laura De Michieli, Alessandro Lupi, Giulio Sinigiani, Angela Tietto, Alessandro Salvalaggio, Antonio Branca, Stefano Da Pozzo, Stefania Rizzo, Diego Cecchin, Martina Perazzolo Marra, Tamara Berno, Domenico Corrado, Chiara Briani, Alberto Cipriani

**Affiliations:** 1 Department of Cardio-Thoraco-Vascular Sciences and Public Health, University of Padua, Via Giustiniani, 2, 35128 Padua, Italy; 2 Cardiology Unit, University Hospital of Padua, 35128 Padua, Italy; 3 Padova Neuroscience Center (PNC), University of Padua, 35128 Padua, Italy; 4 Department of Neurosciences, University of Padua, 35128 Padua, Italy; 5 Ematology Unit, University of Padova, 35128 Padova, Italy; 6 Radiology Unit, University Hospital of Padua, 35128 Padua, Italy; 7 Cardiovascular Pathology, Department of Cardiac, Thoracic and Vascular Sciences and Public Health, University of Padova, 35131 Padova, Italy; 8 Nuclear Medicine Unit, Department of Medicine (DIMED), Azienda Ospedale Università di Padova, 35128 Padua, Italy

**Keywords:** transthyretin amyloid cardiomyopathy, therapy, clinical trials, future treatments

## Abstract

Transthyretin (TTR) amyloid cardiomyopathy (ATTR-CM) is a progressive disease that has emerged as a significant cause of heart failure. Advances in the understanding of ATTR-CM pathophysiology have revolutionised its therapeutic landscape over the past decade, with the development of targeted therapies that are able to improve survival and quality of life. TTR stabilizers, such as tafamidis and acoramidis, can reduce TTR instability and subsequent amyloid fibril formation. Clinical trials have demonstrated their efficacy both in improving survival and quality of life in patients with ATTR-CM. Gene-silencing therapies using small interfering RNAs (siRNAs), such as patisiran and vutrisiran, or antisense oligonucleotide inhibitors (ASOs), such as inotersen and eplontersen, serve as powerful therapeutic options by decreasing TTR production; trials on patients with ATTR-CM have been recently published or are ongoing. Novel, emerging therapies aim to enhance fibril clearance using monoclonal antibodies, such as NI006, that target amyloid deposits in the myocardium, promoting their depletion, plausibly with regression of the structural and functional impairments caused by the disease. Concurrently, advancements in diagnostic modalities have facilitated earlier detection of this disease, allowing the timely initiation of treatment with a more significant impact on patients’ survival and quality of life. Despite these strides, challenges remain, including the high cost of disease-modifying therapy and the need for response criteria to monitor treatment’s efficacy. Future directions will involve improving patients’ screening to achieve earlier diagnoses, optimising patients’ selection for disease-modifying therapy and identifying criteria for the treatment’s response or lack thereof to possibly consider therapy switch or associations. In this review, we will explore the more recent therapeutic advancements in ATTR-CM, starting from traditional heart failure therapies and moving to disease-modifying therapies with a detailed evaluation of the registration trials to explore the strengths and shortcomings of each treatment.

## 1. Introduction

The term amyloidosis encompasses a broad spectrum of infiltrative diseases that arise from the deposition of amyloid fibrils in the extracellular matrix of various organs, including the heart [1]. Although several forms of amyloidosis have been described, almost 95% of patients suffering from amyloid cardiomyopathy present alternatively two forms [2,3]: light chain amyloidosis (AL) or transthyretin amyloid cardiomyopathy (ATTR-CM), which could be hereditary (ATTRv-CM) or wild type (ATTRwt-CM). Whereas the first results from a clonal production of immunoglobulin light chains, most commonly arising from a plasma cell dyscrasia [4,5], the latter occurs when transthyretin (TTR) amyloid fibrils accumulate in myocardial tissue [6,7].

TTR is largely synthesised by the liver and normally circulates as a tetramer, transporting thyroid hormone thyroxine (T4) and retinol-binding protein [8]. In ATTR-CM, mutations in the *TTR* gene (in ATTRv-CM) or age-related yet incompletely characterised mechanisms (in ATTRwt-CM) cause instability of TTR’s tetrameric structure, with progressive dissociation into monomers; these monomers misfold, aggregate into oligomers and amyloid fibrils that then deposit in the interstitial space between cardiac myocytes [7].

The precise mechanisms and risk factors leading to TTR tetramer dissociation into misfolded monomers, which ultimately aggregate into amyloid fibrils, remain incompletely understood. Several factors contribute to TTR destabilisation and favor its dissociation into monomers, including oxidative modifications, age-related decline in cellular homeostasis, metal ions, and genetic mutations. In addition to TTR tetramer dissociation, a proteolytic mechanism contributing to amyloid formation has recently been identified through studies on the Ser52Pro TTR variant [7,8,9].

In ATTR-CM, various mechanisms of tissue damage are present, including chronic infiltration with steric encumbrance and a proteotoxic effect of the amyloid fibrils and precursor proteins [10,11]. This process can lead to diastolic dysfunction with impaired ventricular filling, up to a restrictive physiology, and progressive heart failure (HF), most frequently with preserved ejection fraction (HFpEF) [12,13]. The condition may also be associated with arrhythmias [14,15], autonomic dysfunction [16,17], valvular heart disease [18] and potential thromboembolic complications [19], which together contribute to significant morbidity and mortality.

The incidence of ATTR-CM remains difficult to determine with certainty, but increasing evidence suggests this disease is far more prevalent than previously thought, particularly in high-risk subsets [20].

## 2. Heart Failure Therapy in ATTR-CM

ATTR-CM is a progressive and life-threatening condition increasingly diagnosed in clinical cardiology [21,22], and its recognition at an early stage is of paramount importance, so that treatment options can have an impact both on survival and quality of life.

For years, treatment options for ATTR-CM were limited, focusing primarily on symptomatic therapies and clinical management of HFpEF, namely diuretic therapy [22]. At the present time, it is debatable whether conventional HF medications cause a significant benefit in patients with ATTR-CM, since patients with known cardiac amyloidosis have been excluded from most of the trials investigating such medications [23]. It is worth noting that the pathophysiological and hemodynamic profile of ATTR-CM, as well as the frequent involvement of the autonomic nervous system [6,7], could make the initiation and up titration of the conventional HF medication, such as beta-blockers, renin–angiotensin–aldosterone system (RAAS) inhibitors, and mineralocorticoid receptor antagonists, particularly challenging. However, this might be somewhat of a historical heritage related to the severely restrictive profile of ATTR-CM patients diagnosed in late stages, as it used to happen before the medical and scientific revolution that transformed this field. Recently, Ioannou et al. [23] observed that in a large cohort of patients with ATTR-CM, less than 60% of the overall cohort was treated with beta-blockers and ACE-i/ARB, which were often prescribed at a low dose and discontinued in 22% and 33% of patients, respectively; on the other hand, 39% of patients received MRAs, which were discontinued in 8% of cases during follow-up. With the limitation of a retrospective study with propensity score-matched analysis, the Authors concluded that treatment with an MRA was independently associated with a lower risk of mortality in the overall ATTR-CM population, whereas treatment with a beta-blocker was independently associated with a lower risk of mortality in patients with ATTR-CM and echocardiographic evidence of left ventricular ejection fraction (LVEF) ≤ 40%. This finding is consistent with other studies that have investigated the effects on survival of beta-blocker exposure in patients with ATTR-CM [24,25]. Clearly, these findings must be interpreted with caution due to the lack of randomised trials and the inevitable treatment bias related to the fact that beta-blockers and other HF therapies might be prescribed in less severely affected patients without restrictive hemodynamics.

Lang and colleagues [26] investigated the safety and efficacy of sodium–glucose cotransporter 2 inhibitors (SGLT2i) in a cohort of 87 ATTR-CM patients, mostly wild type, treated with SGLT2is versus 95 untreated control patients. SGLT2i were well tolerated by most patients with ATTR-CM (11.5% of patients discontinued treatment, mostly due to genitourinary symptoms) and they appeared to improve volume status and combat diuretic resistance. Porcari et al. [27] performed a larger retrospective multicentre study to evaluate the use of SGLT2i in patients with ATTRwt-CA and ATTRv-CA; with a propensity matched analysis, treatment with SGLT2i was associated with reduced risk of all-cause mortality, cardiovascular (CV) mortality, HF hospitalisation, and the composite outcome of CV mortality and HF hospitalisation; at 12 months, SGLT2i treatment was associated with less worsening of New York Heart Association (NYHA) class, N-terminal pro–B-type natriuretic peptide (NT-proBNP), kidney function, and fewer new initiations of loop diuretics. Nevertheless, at the present day, there is no available randomised controlled trial investigating the tolerability and clinical benefit of SGLT2i in patients with ATTR-CA.

Currently, no studies have evaluated the use of sacubitril–valsartan or vericiguat in ATTR-CM. While these therapies have undeniably shown benefits in other forms of HF [28,29,30], their safety and efficacy in patients with this disease remain unstudied.

Finally, it is worth noting that, in patients with ATTR-CM, atrial fibrillation (AF) is a frequent comorbidity, particularly in the wild-type form, due to progressive atrial remodelling and amyloid infiltration [31]. The coexistence of AF in this population represents a significant clinical challenge, as it often leads to worsening HF symptoms, increased hospitalisation rates, and heightened thromboembolic risk [31]. A key consideration in the management of AF in ATTR-CM is anticoagulation therapy. Unlike in the general population, where anticoagulation decisions are commonly guided by the CHA_2_DS_2_-VA score, in ATTR-CM patients the utility of this score is limited; several expert consensus and international guidelines recommend anticoagulation in patients with ATTR-CM and AF irrespective of the CHA_2_DS_2_-VA score due to the intrinsic thrombogenic risk associated with amyloid-related atrial dysfunction [32,33].

## 3. Disease-Modifying Therapies in ATTR-CM

**Figure 1:**
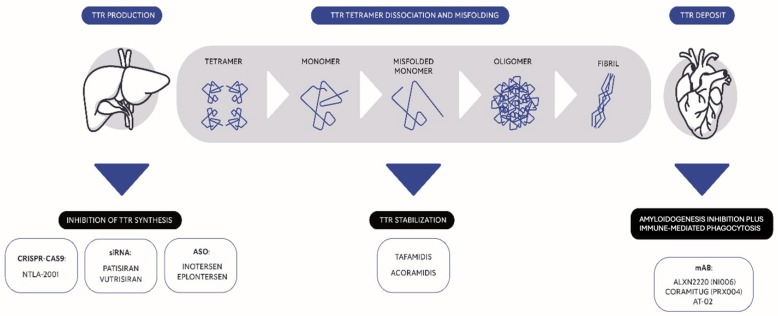
Present therapeutic landscape of transthyretin amyloid cardiomyopathy (ATTR-CM). Current treatment of ATTR-CM aims to silence TTR production, stabilise the TTR tetramer or actively promote clearance of already formed amyloid from TTR deposits. ASO: antisense oligonucleotides. CRISPR-CAS9: clustered regularly interspaced short palindromic repeats CAS9. mAb: monoclonal antibody. siRNA: short interfering ribonucleic acid.

The recent expansion of disease-modifying therapies represents a paradigm shift in the treatment of ATTR-CM, aiming to alter the disease’s natural course. These therapies aim to either stabilise the TTR tetramer, silence TTR production, or actively promote amyloid clearance (Figure 1). In the following chapters, we will analyse the main molecules and clinical trials that are significantly changing the therapeutic landscape of ATTR-CM (Table 1), with a critical focus on their strengths and limitations and subsequent impact on clinical practice.

***(1)***  ***TTR stabilisation***

Two main molecules have been investigated in randomised clinical trials with the purpose of TTR stabilisation: tafamidis and acoramidis. Diflunisal, which is a non-steroidal anti-inflammatory agent acting as a non-selective TTR stabiliser, has been investigated in non-randomised studies with small cohorts of patients and non-negligible adverse effects [34]; it is rarely clinically used nowadays and not reported in recent Consensus Documents and Guidelines [35,36]. The following paragraphs will focus on tafamidis and acoramidis and their respective clinical trials.

*(A)*  *Tafamidis*

The ATTR-ACT (Tafamidis Treatment for Patients with Transthyretin Amyloid Cardiomyopathy) trial [37] was the first pivotal study investigating a disease-modifying therapy specifically in ATTR-CM; previous trials with tafamidis [38] and other molecules, such as inotersen [39] and patisiran [40], focused on ATTR-related neuropathy. The ATTR-ACT trial evaluated tafamidis, a benzoxazole derivative that binds to the thyroxine-binding sites of TTR with very high selectivity and inhibits the dissociation of tetramers into monomers [41]. In this phase III, multicentre, international, double-blind, parallel-design, placebo-controlled trial, 441 ATTR-CM patients were randomised to receive tafamidis (20 mg or 80 mg) or placebo for 30 months. These patients had biopsy-proven (from cardiac and noncardiac sites, such as fat aspirate, gastrointestinal sites, salivary glands, or bone marrow) ATTRwt-CM or ATTRv-CM; the majority were white males with a median age of 75 years. Overall, 25% were affected by ATTRv-CM, with Val122Ile, Thr60Ala, and Ile68Leu being the most common TTR mutations. The primary endpoint included a composite of all-cause mortality and frequency of CV-related hospitalisations (hierarchically assessed according to the Finkelstein–Schoenfeld method), while secondary endpoints assessed change from baseline in functional capacity (measured by distance walked on the 6 min walking test, 6MWT) and quality of life (assessed by the Kansas City Cardiomyopathy Questionnaire, KCCQ [42]). After a 30-month follow-up period, the trial demonstrated that tafamidis significantly reduced all-cause mortality by 30% and CV-related hospitalisations by 32% when compared to placebo. With Kaplan–Meier analysis, the curves for mortality started to diverge after approximately 18 months of treatment. Furthermore, patients receiving tafamidis experienced a slower decline in functional capacity and quality of life, with differences already observed at 6 months. A prespecified analysis showed that the reduction in mortality and functional decline was similar between ATTRwt-CM and ATTRv-CM patients treated with tafamidis [43], although untreated ATTRv-CM patients had the worst prognosis. The open-label extension (OLE) study confirmed that patients initially randomised to placebo had worse long-term survival than those randomised to tafamidis [44], although there was a signal towards improved survival in patients transitioned from placebo to tafamidis during follow-up.

The ATTR-ACT trial represented the first, long-awaited, hope for disease-specific therapy for patients with ATTR-CM. Some limitations of the study must be highlighted, including that the study population was predominantly male and mostly affected by ATTRwt-CM. Secondly and importantly, the study focused on a cohort of patients whose characteristics were typical for the years during which the study was conducted, namely biopsy-proven ATTR-CM with advanced phenotype (with significantly elevated NT-proBNP) and with 30% of patients in NYHA class III. Fortunately, patients currently seen in clinical practice can benefit from a non-invasive diagnostic approach [45] when a monoclonal component has been excluded and are generally diagnosed at earlier stages [20,21,22] with milder phenotypes.

Interestingly, in the ATTR-ACT trial, there was no significant reduction of CV-related hospitalisations among the subgroup of patients with ATTR-CM and NYHA class III who received tafamidis compared to placebo. The authors speculated that the higher hospitalisation rate observed in this group was attributable to longer survival during a more severe phase of the disease, underscoring the importance of early diagnosis and treatment. Elliot et al. [44,46] subsequently analysed the impact of treatment with tafamidis in patients with ATTR-CM who presented advanced HF symptoms, classified as NYHA class III. Although tafamidis significantly enhanced all-cause survival in this group of patients, patients with NYHA class III experienced an increased risk of CV-related hospitalisations, compared to those with lower NYHA classes. This again highlights the importance of a timely diagnosis in patients with ATTR-CM in order to achieve not only a higher survival rate but also a better quality of life.

Currently, tafamidis represents the only approved treatment for ATTR-CM in several countries, making it a cornerstone therapy for clinicians managing this condition. Current real-life experiences are being published [47,48,49,50], analysing the efficacy of tafamidis outside the controlled environment of a clinical trial, involving a more diverse and representative patient group. Debonnaire and colleagues [49] conducted a multicentre international study of 710 ATTRwt-CM patients to investigate the impact of tafamidis treatment in octogenarians and found that, after propensity score matching, this treatment was associated with lower mortality in patients > 80 years old. Neither age at diagnosis nor at treatment initiation interacted with tafamidis’ mortality benefit. Survival was worse despite tafamidis in individuals ≥ 90 years, NAC [51] stage ≥ 3, NYHA class ≥ III. A study from the Transthyretin Amyloidosis Outcomes Survey (THAOS) Registry [48], including both ATTRwt-CM and ATTRv-CM, compared tafamidis-treated and tafamidis-untreated patients and reported better survival in the first group. The survival rate at 30 months in tafamidis-treated patients (84.4%) was higher than that of the treatment arm of ATTR-ACT (70.5%), and a similar trend was noted between tafamidis-untreated patients (70.0%) and the placebo arm of ATTR-ACT (57.1%), underscoring once again the changing landscape of patients affected by ATTR-CM. Clearly, this study is limited by its observational nature, as treated patients had less severe disease, as indicated by lower median NT-proBNP values, than untreated patients and were more likely to have been enrolled in THAOS in 2019 or later. Masri et al. [52] reported a contemporary long-term outcomes analysis of patients with ATTR-CM treated with tafamidis. Among 624 treated patients, 39% died at 43 months and the probability of survival was closely related, among others, to age, NYHA class and disease stage at diagnosis.

Establishing clear criteria to evaluate therapeutic response remains a remarkable challenge. Ioannou et al. [53] suggested the role of an increase in NT-proBNP and outpatient diuretic intensification (ODI) as potential markers to detect disease progression in ATTR-CM. Furthermore, a decline in estimated glomerular filtration rate has been associated with increased risk of mortality [54]. It is noteworthy that these markers, while extremely promising, were derived from patients’ cohorts with only a subset receiving treatment with tafamidis. As such, the applicability of these markers warrants further investigation to validate their relevance and reliability in this specific context.

*(B)*  *Acoramidis*

The ATTRIBUTE (Efficacy and Safety of Acoramidis in Transthyretin Amyloid Cardiomyopathy) trial [55] investigated acoramidis, a TTR stabiliser deemed to achieve almost complete stabilisation in both ATTRwt-CM and in the most common TTR variants [55,56,57]. A rare mutation in the gene encoding TTR, T119M, results in a variant protein characterised by a higher stability of the tetramer compared to wild-type TTR. Acoramidis was designed to mimic the activity of the T119M variant, thus reaching better potency, binding affinity and TTR stabilisation when compared to tafamidis [58]. In this phase III, multicentre, international, double-blind, placebo-controlled trial, 632 ATTR-CM patients were randomised to receive acoramidis 800 mg twice daily or placebo for 30 months. These patients presented alternatively with ATTRwt-CM or ATTRv-CM, which could either be biopsy proven (endomyocardial biopsy) or non-invasively diagnosed with a technetium-99m bone scintigraphy, with a Perugini grade equal to or greater than two and the exclusion of a monoclonal component. Like the ATTRACT trial, the study population was predominantly composed of white males, with a median age of 77 years. Only 10% of those who underwent randomisation were affected by ATTRv-CM, with V122I being the most frequent TTR variant. Treatment with tafamidis was not allowed during the initial 12 months of the trial but was permitted thereafter. The primary endpoint was represented by a four-step primary hierarchical analysis, which included all-cause mortality, frequency of CV-related hospitalisation, the change from baseline in NT-proBNP level, and the change from baseline in the 6MWT. Aside from evaluating functional capacity (via the 6MWT) and quality of life (via the KCCQ) as secondary endpoints, the trial also analysed the change from the baseline of serum TTR levels.

Treatment with acoramidis was associated with a reduced combined risk of all-cause mortality and CV-related hospitalisations when compared to placebo (Finkelstein–Schoenfeld test statistic, 5.015; *p* < 0.001; win ratio of 1.8 [1.4 to 2.2] 95% CI, *p* < 0.05); moreover, acoramidis was effective in slowing disease progression, as evidenced by meaningful improvements in quality of life and functional capacity (the decrease from baseline in the 6MWT had a mean difference of 39 metres between the acoramidis and placebo group, favouring the former), as well as in maintaining favourable serum TTR levels compared to patients receiving placebo. Interestingly, the trial showed no statistically significant difference in all-cause mortality between patients who received acoramidis and those who were given a placebo. This outcome likely reflects the remarkable improvements in the overall care and management of ATTR-CM patients in recent years. To set this in perspective, patients in the ATTRIBUTE trial placebo group showed a better 30-month survival rate than the patients in the ATTR-ACT trial tafamidis group (30-month survival of 74.3% in the ATTRIBUTE placebo group vs. 70.5% in the combined tafamidis treatment groups in the ATTR-ACT trial). The increased awareness of the disease and the enhanced non-invasive diagnostic tools have led to earlier detection, and a more comprehensive supportive care has undeniably contributed to improved survival rates, even for patients who do not receive specific therapies [59]. Consequently, it is more challenging for new additional therapies to demonstrate survival benefits over a placebo in a clinical trial. A report of the first 12 months of the OLE study [60], whose participants were required to discontinue tafamidis, showed a significant difference in all-cause mortality as well as first CV hospitalisation between the continuous acoramidis and placebo-to-acoramidis group; while there may be a trend of reduction in the risk of all-cause mortality in the placebo arm following initiation of acoramidis, further ongoing follow-up will clarify the impact of acoramidis in this subset.

More insights into the mechanisms of clinical benefit of acoramidis were provided by the cardiac magnetic resonance (CMR) substudy [61], which reported that acoramidis trended toward improving or stabilising structural and biventricular functional CMR parameters compared to placebo. Notably, at 30 months, 12.5% of acoramidis recipients demonstrated amyloid regression, defined as a reduction of at least 5% in extracellular volume (ECV), suggesting that TTR stabilisation may allow the rate of innate amyloid clearance mechanisms to exceed the rate of amyloid formation.

***(2)***  ***Reduction in TTR Synthesis***

Another potential target in the pathophysiology of ATTR-CM is the reduction in TTR synthesis. Until recently, orthotopic liver transplantation was considered the only option to interrupt the synthesis of variant TTR in ATTRv patients; however, this is no longer a therapeutic tool in most centers, and it is not an option in ATTRwt-CM [36]. The following paragraphs will focus on contemporary strategies for the reduction in TTR synthesis.

*(A)*  *Antisense oligonucleotide inhibitors*

Antisense oligonucleotide inhibitors (ASOs) are short, synthetic strands of ribonucleic acid (RNA) specifically designed to target a determined messenger RNA, thus halting its translation into protein. This precise mechanism allows ASOs to reduce or completely silence the production of a particular protein, such as TTR.

- *Inotersen*

Inotersen is a 2′-O-methoxyethyl–modified ASO that targets TTR messenger RNA in hepatocytes, thus promoting its degradation through the RNAase H1 pathway, and reducing the overall TTR production [62,63]. The NEURO-TTR [63] (Inotersen Treatment for Patients with Hereditary Transthyretin Amyloidosis) was a phase III, international, randomised, double-blind, placebo-controlled trial analysing the safety and efficacy of treatment with inotersen in patients with ATTRv with polyneuropathy (ATTR-PN). After a 15-month follow-up period, the trial confirmed the efficacy of inotersen treatment in ATTR-PN, as it significantly reduced the neurological manifestation of the disease, along with increasing patients’ quality of life, when compared to placebo. However, there were safety concerns regarding thrombocytopenia and glomerulonephritis, such that platelet count and kidney function require close monitoring during treatment in clinical practice. No significant changes in cardiac structure were documented between patients receiving inotersen compared to placebo. A dedicated trial on patients with ATTR-CM was not pursued by the company; an open-label study [64] reported a trend towards improvement of the cardiac structure and functional capacity in ATTR-CM patients treated with inotersen; however, this included a small cohort of patients (n = 33).

- *Eplontersen*

A next-generation antisense oligonucleotide inhibitor, eplontersen, is currently under investigation for the treatment of ATTR-CM. This molecule is conjugated to a triantennary N-acetyl galactosamine (GalNAc) ligand for enhanced uptake by hepatocytes [65]. The phase 3, open-label, clinical trial for ATTRv patients with PN, NEURO-TTRansform trial, compared eplontersen with the placebo cohort of the NEURO-TTR trial, showing a significantly lowered serum TTR concentration, less neuropathy impairment and better QoL. The ongoing CARDIO-TTRansform trial (NCT04136171) will evaluate, enrolling more than 1400 patients, the impact of treatment with eplontersen in patients with ATTR-CM. This will be the largest ATTR-CM trial to date, and results are expected in 2026.

*(B)*  *Short interfering RNAs*

Short interfering RNAs, or siRNAs, aim to silence liver expression of TTR by targeting the messenger RNA in hepatocytes, thereby reducing TTR levels through an RNA interference mechanism [66,67,68]. By inhibiting the translation of TTR, these agents rapidly knock down its formation and subsequent misfolding, preventing deposition of further amyloid fibrils in the extracellular cardiac matrix [67,68,69].

- *Patisiran*

The APOLLO-A (Patisiran, an RNAi Therapeutic, for Hereditary Transthyretin Amyloidosis) trial [40], evaluated the use of the siRNA patisiran in hereditary ATTR-PN. Treatment with patisiran was associated with significant quality of life improvement and neurological manifestation reduction when compared to placebo. In a prespecified subgroup of patients with ATTR-CM, measures of cardiac structure and function favoured patisiran over placebo at 18 months. The APOLLO-B (Patisiran Treatment in Patients with Transthyretin Cardiac Amyloidosis) trial [70] investigated specifically the use of patisiran in ATTR-CM. In this phase III, multicentre, international, double-blind, randomised trial, 360 ATTR-CM patients were randomised to receive either patisiran 0.3 mg/kg of body weight (maximum dose of 30 mg) intravenously or placebo once every three weeks for 12 months. The study population was composed mostly of ATTRwt-CM (80%), and patients were diagnosed through tissue biopsy or by fulfilling validated non-invasive diagnostic criteria [45]. Other key inclusion criteria were the history of HF and a baseline NTproBNP greater than 300 ng/L (600 ng/L in patients with atrial fibrillation) but lower than 8500 ng/L. Notably, patients with both NYHA III and NAC stage 3 were excluded, as were patients with NYHA IV. Both the patisiran group and the placebo group consisted predominantly of male patients, median age of 76 years; compared to other studies, such as the ATTRIBUTE trial, there was a more consistent percentage of ATTRv-CM (20%) and a greater ethnic diversity within the study population; however, white ethnicity remained the most represented demographic among participants. Interestingly, approximately 25% of patients undergoing randomisation received tafamidis at baseline treatment. Still, the size of the trial population precluded any formal evaluation of the patisiran treatment in this subgroup. The primary endpoint of the APOLLO-B trial was to investigate functional capacity, analysing the change from baseline in the distance walked on the 6MWT. Secondary key endpoints included modifications in quality of life and composite outcomes, including death from any cause, hospitalisations for any cause, and urgent HF visits over 12 months. Exploratory endpoints included changes in cardiac biomarkers and echocardiographic parameters. After a 12-month follow-up period, the trial reported how patisiran preserved functional status, both as 6MWT and KCCQ results; however, the decrease from baseline in the 6MWT had a mean difference of only 14 metres between the placebo and patisiran groups, favouring the latter. There were no statistically significant differences regarding the other secondary endpoints. This outcome may be attributed to the short duration of the trial, which may have been insufficient to capture long-term benefits in a slow progressive disease, such as ATTR-CM. Secondly, as mentioned before for the ATTRIBUTE trial results, the patient population is constantly evolving, and it is progressively more challenging to detect the beneficial impact of targeted therapies.

- *Vutrisiran*

Vutrisiran is a next-generation subcutaneously administered RNA interference agent [68,69]. Vutrisiran siRNA is conjugated to a GalNAc ligand that binds the asialoglycoprotein receptor expressed on the surface of hepatocytes [68,69]. This conjugate provides enhanced stabilisation chemistry, allowing for subcutaneous (SC) injections every 3 months. In the HELIOS-A trial, including patients with ATTRv polyneuropathy, vutrisiran improved disease-relevant outcomes were compared to an external placebo cohort (from APOLLO trial) [71]. An exploratory analysis on cardiac parameters (REF doi:10.1002/ejhf.3138) showed that, at 18 months, vutrisiran was beneficial in terms of NT-proBNP values and some echocardiographic findings in the modified intent-to-treat (mITT) population and a cardiac subpopulation (n = 40). In a planned cohort undergoing bone scintigraphy assessments, most vutrisiran-treated patients experienced reduced or stabilised radiotracer uptake versus baseline.

The HELIOS-B (Vutrisiran in Patients with Transthyretin Amyloidosis with Cardiomyopathy) trial [72] investigated the safety and efficacy of vutrisiran in ATTR-CM. In this phase III, multicentre, international, double-blind, randomised, placebo-controlled trial 655 ATTR-CM patients were randomised to receive either vutrisiran 25 mg subcutaneously once every twelve weeks or placebo for 36 months. The trial population included both ATTRv-CM or ATTRwt-CM patients, with the latter representing approximately 90% of the population. Like the APOLLO-B trial, inclusion criteria required either a biopsy-proven diagnosis of ATTR-CM or the fulfilment of non-invasive diagnostic criteria, and a history of HF with baseline NT-proBNP greater than 300 ng/L (600 ng/L in atrial fibrillation) but lower than 8500 ng/L. Of those who underwent randomisation, about 40% were receiving tafamidis treatment at baseline; for this reason, the results of the trial were, respectively, referred to as the general population and the monotherapy population (patients who were not receiving tafamidis at baseline). The primary endpoint of the HELIOS-B trial was represented by a composite of death from any cause and recurrent CV events, whereas secondary endpoints included death from any cause through 42 months, change from baseline in both functional capacity (measured by distance covered on the 6MWT) and quality of life (assessed by the KCCQ-OS). A subgroup analysis considering baseline disease severities in HELIOS-B (in terms of NYHA class and NTproBNP values) showed that the greatest benefit was achieved in earlier, less severe disease [73].

The HELIOS-B trial marked a pivotal advancement in the therapeutic management of ATTR-CM, as it demonstrated, in a modern cohort, that vutrisiran treatment was associated with lower risk of the composite outcome of death from any cause and recurrent CV events when compared to placebo, both in the overall (HR 0.72; 95% CI, 0.56 to 0.93; *p* = 0.01) and monotherapy population (HR 0.67; 95% CI, 0.49 to 0.93; *p* = 0.02). Furthermore, this benefit was broadly consistent even for all the secondary endpoints, with reduced risk of death from any cause through 42 months when compared to placebo (HR, 0.65; 95% CI, 0.46 to 0.90; *p* = 0.01), reduced decline in functional capacity and quality of life. Similarly encouraging results were observed in the monotherapy population.

The HELIOS-B trial provided decisive data on the impact of vutrisiran in ATTR-CM, not only regarding performance status and quality of life but also on hard clinical endpoints such as hospitalisation and mortality from CV causes in a modern cohort of patients with earlier diagnoses and patients presenting in an overall better health state [22,74,75]. Compared to the ATTR-ACT trial population, the HELIOS-B patients presented generally a less severe disease at baseline, according to NYHA class (29% of NYHA class III patients in the ATTR-ACT population vs. 8% of NYHA class III patients in the HELIOS-B population), KCCQ overall score (mean value of 67 in the ATTR-ACT population vs. mean value of 73 in the HELIOS-B population), distance covered on 6MWT (mean value of 350 metres in the ATTR-ACT population vs. mean value of 377 metres in the HELIOS-B population), and NT-proBNP (mean value of 2995 ng/L in the ATTR-ACT population vs. mean value of 1801 ng/L in the HELIOS-B population), and could potentially benefit from a more robust arsenal of therapies, such as tafamidis or HF therapies. Despite these evolving population dynamics, vutrisiran has demonstrated clear efficacy over placebo, highlighting its potential as an effective treatment option even for patients at earlier disease stages. The favourable safety profile and relatively infrequent dosing schedule of vutrisiran make it especially suitable for long-term use, further supporting its application in early-stage patients.

At present day, patisiran has not received Food and Drug Administration (FDA) authorisation for ATTR-CM treatment due to the limited treatment effects on 6MWT and KCCQ and the lack of documentation of improved all-cause mortality and CV events. Treatment with vutrisiran for ATTR-CM is now approved by the FDA and is currently under consideration by the European Medical Agency (EMA).

*(C)*  *Gene editing*

The clustered regularly interspaced short palindromic repeats and associated Cas9 endonuclease (CRISPR-Cas9) system is a Nobel-winning technology that has recently emerged as a revolutionary tool in the treatment of ATTR-CM; ATTRv-CM was the first disease treated in vivo with this method [76]. By enabling precise genetic editing, CRISPR-Cas9 targets the root cause of the disease at a genetic, DNA level. The CRISPR-Cas9 system has been applied to cleave the mutated TTR gene in hepatocytes, the primary source of TTR production, thereby preventing its translation and subsequent protein formation.

Gillmore et al. [76] have evaluated the safety and efficacy of in vivo CRISPR-Cas9 gene editing in humans with ATTRv with PN. In this phase I, open-label, multicentre study, six patients were given a single dose of NTLA-2001, a CRISPR-Cas9 system targeting TTR gene, at a dosage of either 0.1 mg/kg or 0.3 mg/kg. The authors demonstrated a significant reduction (up to 87%) in serum TTR levels compared to baseline at only 28 days of follow-up. No serious adverse events were observed in the study population. These findings provide strong evidence for the potential of CRISPR-Cas9 to achieve sustained therapeutic effects with only one infusion, representing a paradigm change when compared to other chronic treatments, such as TTR stabilisers or silencers. In the expanded phase 1 trial enrolling 36 patients receiving nexiguran ziclumeran (nex-z, also known as NTLA-2001) [77], infusion-related reactions (in five patients) and transient elevations in AST levels (in two patients) were reported. Serious adverse events (most of which were consistent with ATTR-CM) were reported in 14 patients. Only one of these events was considered to be related to nex-z (serious infusion reaction). A single dose of this compound was associated with deep and durable TTR concentration reductions, as well as limited disease progression during the first 12 months of treatment. The ongoing phase III MAGNITUDE trial (NCT06128629) is investigating the efficacy and safety profile of NTLA-2001 in patients with ATTR-CM.

Nonetheless, uncertainties about the long-term effects of such gene-editing therapy remain. Potential risks include immune responses against the Cas9 enzyme or reactivation of the TTR gene due to natural hepatocyte turnover; these concerns will be addressed through extended clinical follow-up, which will be of paramount importance in establishing the long-term safety profile of NTLA-2001 and its viability as a targeted therapy for ATTR-CM.

***(3)***  ***Amyloid deposits removal***

Monoclonal antibodies (mAbs) represent a fascinating novelty as potential therapies in ATTR-CM, as they display a new, distinct pharmacodynamic: amyloid clearance. The deposition of amyloid fibrils is not to be considered as a static process, but rather a dynamic interplay between two different mechanisms: formation of misfolded TTR monomers/oligomers/fibrils with subsequent infiltration, and amyloid clearance [1,3,7,10]. mAb directly targeting amyloid fibrils within the myocardial tissue can promote active clearance through activation of innate immunity. Unlike stabilisers and silencers, which address amyloidogenesis upstream, mAbs aim to reduce the established amyloid burden, potentially halting and reversing disease progression.

- *ALXN2220 (NI006)*

Garcia-Pavia et al. [78] recently investigated the safety of a recombinant human anti-ATTR monoclonal antibody, NI006, in ATTR-CM. NI006 is a recombinant human anti-ATTR monoclonal IgG1 antibody, generated through comprehensive immune repertoire analyses of memory B-cell complement obtained from healthy elderly [79]. NI006 selectively binds ATTRv and ATTRwt deposits, not physiologically folded TTR, and can promote ATTR depletion by inducing antibody-mediated phagocytosis of ATTR fibrils. In the phase I, double-blind, placebo-controlled, ascending dose, randomised trial, 40 ATTR-CA patients were randomised in a 2:1 ratio to either receive intravenous infusions of NI006 (with ascending dose from 0.3 mg/kg to 60 mg/kg of body weight) or placebo every four weeks for a 4-month period. The 4-month placebo-controlled, ascending-dose phase was followed by an 8-month OLE phase in which all participating patients received NI006 with stepwise increases in the dose. Almost all patients enrolled in the study were male, with a median age of 72 years. ATTRwt-CM accounted for more than 80% of the entire population. Concomitant treatment with tafamidis was allowed, but treatment with other ATTR-specific drugs was not permitted. NI006 successfully achieved the primary endpoint of safety, as patients who received NI006 did not experience apparent drug-related serious adverse events. Most frequently, adverse events were HF and arrhythmias (expected complications in ATTR-CM) and musculoskeletal events, mainly arthralgias and arthritis; three patients experienced cytokine release syndrome with an associated increase in cardiac biomarker levels, and two patients had transient, asymptomatic decreases in the platelet count. Interestingly, the authors described a significant reduction in cardiac tracer uptake at bone scintigraphy in patients receiving high doses of NI006 compared to placebo, with a distinct difference already visible at 4 months, and a consistent further reduction at 12 months, also in patients switched to NI006 during the OLE phase. Similar results were achieved in terms of ECV reduction on cardiac MRI and of cardiac biomarkers, such as NT-proBNP and high-sensitivity troponin T (although available in a subset of patients). These conclusions are crucial, as they suggest the efficacy of NI006 in targeting the amyloid deposits already present in the myocardium, therefore reducing the already established disease burden, potentially with reversion of the structural and functional impairment.

The ongoing phase III DEPLETE-TTR trial (NCT06183931) is designed to investigate the safety and efficacy of ALXN2220 (new name for NI006) in patients with ATTR-CM, either ATTRwt-CM or ATTRv-CM, with an NTproBNP > 2000 ng/L and a history of HF; in case of positive results, the mAb approach could truly revolutionise the therapy for patients with ATTR-CM. Indeed, while TTR stabilisation and silencing therapies can slow down or prevent disease progression, this approach could potentially lead to a regression of the ongoing process and a functional recovery of the cardiomyopathy.

- *Other mAbs*

PRX004, now known as coramitug, is another humanised mAb designed to deplete TTR amyloid deposits via antibody-dependent phagocytosis and to inhibit fibril formation by binding misfolded TTR. The phase I clinical trial was recently published [80], including 21 ATTRv patients; the drug was well tolerated and, in seven patients, global longitudinal strain and neurological impairment were stable/slightly improved. Other phase I and phase II clinical trials (NCT05521022/NCT05951049) are ongoing for the pan-amyloid fibril-depleting AT-02.

## 4. Summary and Conclusions

The therapeutic advances discussed in this review illustrate the shift from symptomatic management to targeted, disease-modifying treatments in ATTR-CM. Several studies have been published and are ongoing, exploring molecules that target different aspects of the disease’s pathway. Even with methodological dissimilarities, the different studies have underlined the importance of early diagnosis and timely treatment. Moreover, the outcomes assessed—encompassing both hard endpoints and measures of functional capacity and quality of life—have explored the full potential benefits that these therapies have on patients’ lives.

Thanks to animal models and basic science studies, researchers are gaining deeper insight into the mechanisms of amyloid fibril formation, as well as potential intrinsic and therapeutic pathways for the removal of deposited fibrils [9,81]. Alternative and complementary pathogenic pathways are also being explored, such as the role of plasmin and amyloid cleavage, as seen in the TTR Ser52Pro mutation [9].

Since disease-modifying treatments are most effective in early stages, a timely recognition of ATTR-CM is of paramount importance. This relies heavily on adopting a “cardiomyopathy mindset” [82], a clinical approach that encourages heightened suspicion of infiltrative or rare cardiomyopathies in appropriate settings. Clinicians must remain alert to “red flags”, such as disproportionate cardiac hypertrophy with low voltage on ECG, unexplained HFpEF, bilateral carpal tunnel syndrome or family history of amyloidosis.

The management of ATTR-CM has undergone a remarkable transformation in recent years. Nonetheless, on the one hand, these advancements have undeniably improved patients’ outcomes, on the other hand, the high costs associated with these therapies have brought pharmacoeconomic considerations to the forefront. In public healthcare systems, resource allocation must consider the cost–benefit balance of each prescribed therapy. Conversely, in privately funded systems, access to therapies may be influenced by insurance coverage and patient out-of-pocket expenses, potentially limiting affordability for many individuals. To optimise outcomes while addressing economic constraints, a patient-tailored approach and a timely diagnosis are crucial. Several studies [83,84] have highlighted how advanced ATTR-CM stages are often associated with significant healthcare costs, as patients more often require intensive inpatient care. An early, correct diagnosis, along with personalised therapy tailored to the patient’s specific disease characteristics and genetic profile, could enhance therapeutic efficacy and minimise unnecessary expenses.

As our understanding of ATTR-CM deepens, the future of treatment will, in fact, most likely involve an individualised approach, balancing patient characteristics, disease stage, and comorbidities. Further studies designed to evaluate disease progression and therapy response criteria are necessary to properly tailor treatment and, possibly, consider therapy switches or associations. Moreover, ATTRv is a multisystemic disease, and data on multisystemic responses to treatment (for example, neuropathy) will be of primary importance in future clinical real life. Continuing research, both in controlled trials and real-world settings, will be crucial to refining these therapies and expanding access to effective treatments. With the growing body of evidence and the emergence of innovative drugs, there is renewed hope for improving survival, functional status, and quality of life in patients with ATTR-CM.

**Table 1:** Published and ongoing phase III clinical trials involving specifically patients with transthyretin amyloid cardiomyopathy (ATTR-CM). ATTRwt-CM: wild-type transthyretin amyloid cardiomyopathy. ATTRv-CM: variant transthyretin amyloid cardiomyopathy. ASO: antisense oligonucleotides. CRISPR-CAS9: clustered regularly interspaced short palindromic repeats CAS9. mAb: monoclonal antibody. NYHA: New York Heart Association. siRNA: short interfering ribonucleic acid.

Drug Name	Pharmacological Class	Reference Clinical Trial	Population Characteristics	Effect on ATTR-CM	Side Effects
Tafamidis	TTR Stabilizer	ATTRACT [37] trial	441 patients with ATTRwt-CM or ATTRv-CM, NYHA class I-III.	Reduced hierarchical endpoint of mortality and cardiovascular-related hospitalizations. Slower decline in functional capacity and quality of life.	Mild gastrointestinal side effects (constipation, nausea, diarrhea).
Acoramidis	TTR Stabilizer	ATTRIBUTE [55] trial	632 patients with ATTRwt-CM or ATTRv-CM, NYHA class I-III.	Reduced combined risk of all-cause mortality and cardiovascular-related hospitalizations. Slower decline in functional capacity and quality of life.	Mild gastrointestinal side effects (constipation, nausea, diarrhea). Mild musculoskeletal symptoms.
Patisiran	siRNA	APOLLO-B [70] trial	360 patients with ATTRwt-CM or ATTRv-CM, NYHA class I-III.	Slower decline in functional capacity and quality of life.	Infusion-related reaction, vitamin A deficit.
Vutrisiran	siRNA	HELIOS-B [72] trial	655 patients with ATTRwt-CM or ATTRv-CM, NYHA class I-III. Approximately 40% under tafamidis treatment	Reduced composite outcome of death and recurrent cardiovascular events. Slower decline in functional capacity and quality of life.	Infusion-related reaction, mild musculoskeletal symptoms.
Eplontersen	ASO	CARDIOTTRansform trial (ongoing)	Approximately 1400 patients with ATTRwt-CM or ATTRv-CM, NYHA class I-III.	Sustained reduction in TTR levels. Improved measures of cardiac structure (data from phase I trial [65])	Infusion-related reaction, mild gastrointestinal symptoms (data from phase I trial [65]).
NTLA-2001	Gene Editing Therapy (CRISPR-Cas9 system)	MAGNITUDE trial (ongoing)	Approximately 765 patients with ATTRwt-CM or ATTRv-CM	Sustained reduction in TTR levels (data from phase I trial [76,77]).	Infusion-related reaction, mild gastrointestinal symptoms (data from phase I trial [76,77]).
NI006	Anti-ATTR monoclonal IgG1 antibody	DEPLETE-TTR trial (ongoing)	Approximately 1005 patients with ATTRwt-CM or ATTRv-CM, NYHA class II-IV	Potential amyloid fibril clearance with tracer uptake reduction in bone scintigraphy (data from phase I trial [78,79]).	Infusion-related reaction, mild musculoskeletal symptoms (data from phase I trial [78,79]).

## List of Abbreviations

6MWT	6 min walking test
ASO	antisense oligonucleotides
ATTR-CM	Transthyretin amyloid cardiomyopathy
AL	light chain amyloidosis
CRISPR-CAS9	clustered regularly interspaced short palindromic repeats CAS9
HFpEF	heart failure with preserved ejection fraction
KCCQ	Kansas City Cardiomyopathy Questionnaire
mAb	monoclonal antibody
NYHA	New York Heart Association
siRNA	short interfering ribonucleic acid
